# Biodiversity, traditional medicine and public health: where do they meet?

**DOI:** 10.1186/1746-4269-3-14

**Published:** 2007-03-21

**Authors:** Rômulo RN Alves, Ierecê ML Rosa

**Affiliations:** 1 Departamento de Biologia, Universidade Estadual da Paraíba, Av. das Baraúnas, 351/Campus Universitário, Bodocongó, 58109-753, Campina Grande-PB, Brazil; 2 Departamento de Sistemática e Ecologia, Universidade Federal da Paraíba, 58051-900 João Pessoa, PB, Brazil

## Abstract

Given the increased use of traditional medicines, possibilities that would ensure its successful integration into a public health framework should be explored. This paper discusses some of the links between biodiversity and traditional medicine, and addresses their implications to public health. We explore the importance of biodiversity and ecosystem services to global and human health, the risks which human impacts on ecosystems and biodiversity present to human health and welfare.

## Background

Traditional medicine (TM) is a comprehensive term used to refer both to systems such as traditional Chinese medicine, Indian ayurveda and Arabic unani medicine, and to various forms of indigenous medicine. In countries where the dominant health care system is based on allopathic medicine, or where TM has not been incorporated into the national health care system, TM is often termed "complementary", "alternative" or "non-conventional" medicine [1]. The links between TM and biodiversity are exemplified by a long tradition of healing powers associated with the earth's natural systems, whether this entails medicinal plants and animal species, the ambient salubrious air, spring water or the natural scenery. The pharmacopoeia of folk seties as well as professional medical systems like Chinese, Ayurvedic, Unani and biomedicine contain thousands of medicines made from leaves, herbs, roots, bark, animal, mineral substances and other materials found in nature [2,3].

The interconnections between TM and the biotic environments may be seen in the health benefits derived from the existence of a full complement of species, intact watersheds, climate regulation and genetic diversity, as well as through our fundamental needs for food, water, clean air, shelter and relative climatic constancy [4]. Discussions of the links between TM and biodiversity therefore are imperative [5], particularly when considering the importance of the importance of former as a source of primary health care to 80 percent of the world's population [6].

Connections between environmental and human health have been addressed by authors such as McMichael and Beaglehole [7], who remarked that a) the sustained good health of populations requires enlightened management of our social resources, economic relations, and of the natural world, and b) that many of today's public-health issues have their roots in the same socioeconomic inequalities and imprudent consumption patterns that jeopardize the future sustainability of health. In the same context, Lebel [8] argued that the biomedical approach to health is based on methods of diagnosing and treating specific pathologies: one pathogen = one disease, an approach that does not take into account the connections between disease and socioeconomic factors such as poverty and malnutrition, and even less of the connections between disease and the environment in which sick people live.

Among several avenues to be explored on the links between TM and biodiversity, this paper addresses the importance of biodiversity and ecosystem services to global and human health, the risks which human impacts on ecosystems and biodiversity present to human health and welfare, and the need to stimulate greater awareness among policy makers and the wider public.

## Environmental Degradation and Human Health

The interrelationships between society and nature, and the importance of environmental health to human health, have recently become widely acknowledged [4], and have drawn attention to the fact that biodiversity loss can have indirect effects on human well-being as well. By disrupting ecosystem function, biodiversity loss leads to ecosystems that are less resilient, more vulnerable to shocks and disturbances, and less able to supply humans with needed services. The damage to coastal communities from floods and storms, for example, increases dramatically following conversion of wetland habitats, as the natural protection offered by these ecosystems including regulation of water run-off is compromised. Recent natural disasters in Asia and North America serve to underline this reality [9].

Human health cannot be considered in isolation, for it depends highly on the quality of the environment in which people live: for people to be healthy, they need healthy environments. Agenda 21, which the governments of 185 countries adopted at this conference in Brazil, clearly spelled out the close link between human health and the environment; it also and highlighted the connection between poverty and underdevelopment on the one hand, and the connection between environmental protection and natural resource management on the other [8].

The implications of biodiversity loss for the global environment have been widely discussed, but only recently has attention been paid to its direct and serious effects on human health. Health risks are no longer merely a result of localized exposures to "traditional" forms of pollution – although these still certainly exist. They are also a result of broader pressures on ecosystems, from depletion and degradation of freshwater resources, to the impacts of global climate change on natural disasters and agricultural production. Like more traditional risks, the harmful effects of the degradation of ecosystem services are being borne disproportionately by the poor. However, unlike these more traditional hazards, the potential for unpleasant surprises, such as emergence and spread of new infectious diseases, is much greater [4].

Biodiversity loss diminishes the supplies of raw materials for drug discovery and biotechnology, causes a loss of medical models, affects the spread of human diseases, and threatens food production and water quality [10]. Its reduction has direct effects on the discovery of potential medicines.

Two examples of recently developed drugs, one from a plant and one from an animal, deserve mention. The story of taxol and the Pacific yew illustrates how we may be losing new medicines before species have been analyzed for their chemical content. The commercially useless Pacific yew was routinely discarded as a trash tree during logging of old growth forests in the Pacific northwest region of the United States until it was found to contain the compound taxol, a substance that kills cancer cells by a mechanism unlike that of other known chemotherapeutic agents: it prevents cell division by inhibiting the disassembly of the mitotic spindle [11]. The discovery of the complex molecule taxol and its novel mechanism of action has led to the synthesis of several taxol-like compounds that are even more effective than the natural compound (11, 12), which illustrates how a clue from nature can lead to the discovery of a new class of drugs that would have been extremely difficult to discover in the laboratory. Early clinical trials revealed that taxol was able to induce remission in cases of advanced ovarian cancer unresponsive to other treatments McGuire et al. [13], subsequent experience has shown that taxol may be one of the most promising new drugs available for the treatment of breast and ovarian cancer [12].

The other example that deserves mention is the peptide compounds in the venom of cone snails, a genus of predatory snails numbering about 500 species that inhabit tropical coral reefs. The diversity of these compounds is so great that it may rival that of alkaloids in higher plants and secondary metabolites in microorganisms [14]. Some of these peptide compounds, which have been shown to block a wide variety of ion channels, receptors and pumps in neuromuscular systems, have such selectivity that they have become important tools in neurophysiological research and may become invaluable to clinical medicine. One voltage-sensitive calcium-channel blocker, omega-conotoxin, binds with enormous specificity to neuronal calcium channels and has been found to have potent activity in animals both as an analgesic [15] and as a means of keeping nerve cells alive following ischemia [16].

It is now being studied in advanced clinical trials in its synthetic form (SNX-111, or ziconotide) for the prevention of nerve cell death following coronary artery bypass surgery, head injury and stroke, and for the treatment of chronic, intractable pain associated with cancer, AIDS and peripheral neuropathies [17] SNX-111 has 1000 times the analgesic potency of morphine but, unlike morphine, does not lead to the development of tolerance or addiction or to a clouding of consciousness [18]. As coral reefs are increasingly threatened in many parts of the world [19], the existence of reef-dwelling organisms such as cone snails is similarly threatened.

In addition to the role biodiversity plays in helping people recover from illness, it also makes a significant contribution in preventing disease and illness, since well-functioning ecosystems can help protect human health. It is known that the poor suffer most from scarce or polluted water and air, and from diseases associated with disrupted ecosystems. One critically important service is the role ecosystems play in controlling the emergence and spread of infectious diseases by maintaining equilibria among predators and prey, and among hosts, vectors and parasites in plants, animals and humans. This protective function of biodiversity has only recently begun to be appreciated [20-23].

Examples of human infectious disease that can be affected by upsetting these equilibria include malaria and leishmaniasis through deforestation [24]; Lyme disease through changes in the number of acorns and in the populations of black-legged ticks, white-footed mice and white-tailed deer [25]; Argentine hemorrhagic fever through the replacement of natural grasslands with corn monoculture [26]; and cholera through increased algal blooms, secondary in part to warming seas and to fertilizer and sewage discharge [27].

Issues involving the current scale of human-induced changes to the biosphere and the risks of systemic dysfunction are not yet prominent or well understood within population health research circles. Yet it is a reasonable expectation that this ongoing impairment of Earth's life-support functions poses substantial risks to human health [28]. Also, disturbances reduce the abundance of some organisms, cause population growth in others, modify the interactions among organisms, and between them and their physical and chemical environments. The sheer diversity of human infectious agents and resulting diseases makes it difficult to generalize about the ways in which ecosystem disturbances and changes in biodiversity may influence human health. Nevertheless, some common patterns exist, and some general principles are beginning to be identified [10].

Human activities are known to be crucial to transmission of some diseases. Forest clearance eliminates species that breed in water in tree holes (e.g., the forest *Aedes *species that transmit yellow fever) but provides favorable conditions for those that prefer temporary ground pools exposed to full sunlight (e.g., many of the *Anopheles *species that transmit malaria). Drainage of wetlands eliminates the marshy pools exploited by many species but can provide the open channels preferred by others (e.g., some important European vectors of malaria, and *Culex tarsalis*, a vector of St. Louis encephalitis) [29].

Bell et al. [30] remarked that one of the major lessons from SARS is that the underlying roots of newly emergent zoonotic diseases may lie in the parallel biodiversity crisis of massive species loss as a result of overexploitation of wild animal populations and the destruction of their natural habitats by increasing human populations. They also pointed out that to address these dual threats to the long-term future of biodiversity, including man, a less anthropocentric and more interdisciplinary approach to problems which require the combined research expertise of ecologists, conservation biologists, veterinarians, epidemiologists, virologists, as well as human health professionals is needed.

## Biodiversity and TM

Wild populations of numerous species are overexploited around the globe, the demand created by the traditional medicine being one of the causes of the overexploitation. In this context, research opportunities should focus both on the documentation of the traditional uses of animal and plants in TM and the cultural and ecological aspects associated with such practices [6].

It is quite clear that the practice of TM is not immuned to the current environmental crisis facing our planet. Significant changes in forests, savannas and other vegetational types have impacted on the procurement and preparation, as well as the cost of plant medicine. Desecration of spiritual spots, sacred spaces, and grooves has tended to reduce the dignity of such 'landscapes' and to encourage their abuse [5]. Over the last three decades, forest degradation in the Brazilian Amazon has diminished the availability of some widely used medicinal plant species. Degradation of Amazonian forests may signify not only the loss of potential pharmaceutical drugs for the developed world but also the erosion of the sole health care option for many of Brazil's rural and urban poor [31].

Under the impact of industrialization and urbanization, western medicine has displaced indigenous medical systems in many areas, in the process leaving many without any health care. Traditional medicinal knowledge is rapidly disappearing, owing to cultural change and declining access -in both urban and rural areas- to sources of natural medicinal products. Most villages in the world are no longer surrounded by the natural habitat that formerly served as a medicine cupboard, and bodies of folk knowledge that have accumulated and been honed for thousands of years are disappearing at an alarming rate. In some cases this loss may actually confer net health benefits; but modern society will never know what effective medicinal treatments are being lost [32]. In Latin America, for example, despite the many individual efforts of the governments to preserve the biodiversity for future generations, traditional knowledge, especially that of derived from traditional medicine such as indigenous knowledge, is also disappearing [33].

Transformation of local ecosystems wrought through human economic activities has been exercising severe constraints on the availability and accessibility of specific types of plant and animal species used for medicinal purposes. As forests are degraded into savanna, savanna to scrublands and bushes, and scrublands to desert characteristics in many parts of the Third world, certain species of plants are disappearing altogether. Such a situation poses problems for the future practice of indigenous medicine; with a few exceptions, all medicines are made from concoctions prepared with plants, plant organs or their secreted products [34].

The procurement of plant and animal species needed by indigenous medical practitioners currently requires long distance travel. This affects not only operational costs of providing traditional medical services particularly in urban areas, but also the forms of herbal medicine prepared. For example, freshly prepared herbal medicines are increasingly being replaced by different concoctions, tinctures and powdered forms even in rural areas in order that they can be stored for longer periods without losing their potency or getting spoiled [35].

Despite the importance of TM for public health in many parts of the world, like the current spasm of plant and animal species extinction, as remarked by [5], the practitioners of ethnomedicine (especially herbalists and cult healers) appear to be at a greater risk of extinction than even forests and other biomes. Knowledge of the use of plants is disappearing faster than the plants themselves. The destruction of tropical forests has meant, in many parts of the tropical region, increasing disappearance of native peoples who have been living in these areas and who have accumulated a compendium of folk knowledge about the usefulness of plants for curing various diseases.

On-site communities with extensive knowledge of local environments which may be used towards collaborative conservation and management. Examples of such practices can be found at the Mamirauá Sustainable Development Reserve, Brazil [36,37], in Zimbabwe [38], in the Philippines [39], and in Pacific Islands [40].

Exclusion of local communities from the consultation/decision processes, on the other hand, may lead to the construction of public policies devoid of historical information, and without much social resonance.

## Plants and Animals as Bioresources

Plants and animals have been used as a source of medicines from ancient times [41-43], and even in modern times, animal and plant-based systems continue to play an essential role in health care [10]. Wild and domestic animals and their by-products (e.g., hooves, skins, bones, feathers, tusks) form important ingredients in the preparation of curative, protective and preventive medicine [44]. Additionally, a significant portion of the currently available non-synthetic and/or semi-synthetic pharmaceuticals in clinical use is comprised of drugs derived from higher plants [45,46], followed by microbial, animal and mineral products, in that order [47].

The value of biodiversity to human health has been highlighted in literature [48], one of its most obvious benefit being the large proportion of the pharmaceutical armamentarium that is derived from the natural world. Over 50% of commercially available drugs are based on bioactive compounds extracted (or patterned) from non-human species [49], including some lifesaving medicines such as cytarabine, derived from a Caribbean sponge, which is reputed as the single most effective agent for inducing remission in acute myelocytic leukemia [50]. Other examples of drugs from biological sources include: quinidine to treat cardiac arrhythmias, D-tubocurarine to help induce deep muscle relaxation without general anesthetics, vinblastine to fight Hodgkin's disease, vincristine for acute childhood leukemias, combadigitalis to treat heart failure, ranitidine to fight ulcers, levothyroxine for thyroid hormone replacement therapy, digoxin to treat heart disease, enalapril maleate to reduce high blood pressure, and even aspirin [51,52].

A great number of these natural products have come to us from the scientific study of remedies traditionally employed by various cultures, most of them being plant-derived [53]. It is widely accepted that folk or traditional medicinal uses (ethnomedical information) of plants indicate the presence of a biologically active constituent(s) in a plant. In other words, folk or traditional medicinal uses represent 'leads' that could shortcut the discovery of modern medicines. In fact, the results presented in an often cited work [54] revealed that from 119 known useful plant-derived drugs, 74% of the chemical compounds used as drugs have the same or related use as the plants from which they were derived. As pointed out by that same author, although the results do not mean that 74% of all medical claims for plants are valid, they surely point out that there is a significance to medicinal folklore that was not previously documented. Other papers on this subject [55-58] also attest to the important role of the traditional medicinal use of plants in modern drug discovery.

There has been increasing attention paid to animals, both vertebrates and invertebrates, as sources for new medicines [10]. Animals have been methodically tested by pharmaceutical companies as sources of drugs to the modern medical science [59], and the current percentage of animal sources for producing essential medicines is quite significant. Of the 252 essential chemicals that have been selected by the World Health Organization, 11.1% come from plants, and 8.7% from animals [60]. Of the 150 prescription drugs currently in use in the United States of America, 27 have animal origin [61].

One excellent example of successful drug development from a component of snake venom (*Bothrops jararaca *[Wied 1824]) is that of the inhibitors of angiotensin-converting enzyme (ACE). This enzyme is responsible for converting an inactive precursor into the locally active hormone angiotensin, which causes blood vessels to constrict and hence raises blood pressure [62]. Other excellent example is the work initially conducted by Daly during the 1960s of the skin secretions of dendrobatid frogs from Ecuador, and of other "poison dart" frog species in Central and South America. This work has led to the identification of a number of alkaloid toxins that bind to multiple receptors in the membranes of nerve and muscle cells. One compound derived from these studies, which binds to nicotinic acid receptors associated with pain pathways, the synthetic ABT 594 (Abbott Laboratories), is in Phase II clinical trials, and has generated a great deal of interest, as it has been shown to be 30–100 times more potent as an analgesic than morphine [10]. The marine environment is a rich source of biologically active natural products of diverse structural types, many of which have not been found in terrestrial sources [63]. The sponge *Luffariella variabilis *(Poléjaeff, 1884) produces relatively large amounts of a chemical with anti-inflammatory activity known as monoalide. It was found that monoalide inhibits the action of an enzyme called phospholipase A_2_. The powerful immunosuppressive agent discodermolide originates from another sponge, *Discoderma *sp. [64].

Ingredients sourced from wild plants and animals are not only widely used in traditional medicines, but are also increasingly valued as raw materials in the preparation of modern medicines and herbal preparations. Greater demand and increased human populations are leading to increased and often unsustainable rates of exploitation of wild sourced ingredients, with some wild species already threatened with extinction [65].

Commoditization of plant medicine and animal parts was an insignificant aspect of the practice of TM. In the last few decades, however, there has been a marked increase in the sale of herbal remedies, precipitating large-scale harvesting of medicinal plants, factory-like production of herbal drugs, and animal poaching in many parts of developing countries. Most medicinal plants are gathered from the wild, and countries like India and China reportedly harvest 90 per cent and 80 per cent of their medicinal plants respectively from uncultivated sources [66]. A similar situation exists in Africa, where due to ever-expanding populations and the expansion of practices such as logging, the biodiversity dependent communities are currently facing the degradation of the ecosystems on which they depend [67]. Wild populations of species like the pygeum (*Prunus Africana*) and the yohimbe (*Pausinystalia yohimbe*) are currently harvested in unsustainable and destructive ways in order to feed international markets. Around 200 medicinal plant species have been added to the Convention on International Trade in Endangered Species of Wild Flora and Fauna (CITES) appendices [68]. A WWF (World Wide Fund for Nature) report estimates that over two thirds of the 50,000 medicinal plants in use today are still harvested from the wild, from which 4,000–10,000 may now be endangered [69].

In addition to the loss of medicinal plant species, the worldwide market for animal parts and their medicinal derivatives is contributing to loss of some species. The increased use of medicinal animals has led to over-exploitation of species like rhinos, tigers, musk deer, bears, monkeys and pangolins. In spite of international regulations and several national laws against poaching and heavy penalties for culprits, the extremely high prices offered for the parts of some species serve as strong incentives for illegal trade in animal parts to flourish.

Examples of unsustainable harvesting of biological resources as remedies have prompted, among other things, discussions about the use of native *versus *domestic species, primary *versus *secondary forest. For instance, results of studies conducted in tropical forested regions of Brazil differ regarding the relative medicinal importance of primary versus secondary forest and weeds. Indigenous groups [70-72] use more primary forest species than do non-indigenous rural Brazilian communities, which show greater use of secondary vegetation, cultivated plants, and weed species [73-75]. This difference may reflect environmental degradation, increased availability of secondary and weed species, and inaccessibility of primary forests [75]. It may also reflect the fact that weeds, for both biochemical and bioecological reasons, represent a significant part of traditional pharmacopoeias throughout the world [76] and that disturbed vegetation may constitute a preferred habitat for collectors and users of medicinal plants [75].

Many medicinal plant species have spread globally both via intentional and carefully planned transfers and as the unintentional outcome of people's movements [77-79]. According to Voeks [79], the high proportion of medicinal plant species with wide distributions can be interpreted as the result of past and present plant movements resulting in improved medicinal floras around the world. The use of a different domestic animal species as remedies [41,80] suggests that Voeks [79] interpretation is equally applicable to medicinal animals.

The therapeutic indications of wild animals and plants and domestic or cultived species also overlapped in many cases. This aspect opens a perspective of, where suitable, replacing the use of threatened species with others in traditional medicine recipes. Such replacement of products is of interest from a conservationist perspective, in the context of reducing the pressure on overexploited populations, or legally protected species. However, replacement of ingredients in remedies should be done with caution, because as pointed by Sodeinde and Soewu [81], substitutes may not always be feasible because recipes using different species may not have the same efficacy, nor may it be advisable without a thorough examination into the sustainability of utilizing substitute species to ensure the viability of any such exploitation. Additionally, consumers sometimes prefer wild versions. Precaution should also be taken when suggesting the replacement of animal products with plants to ensure the survival of the medicinal animal species. In Brazil, for example, state lists of endangered plants include 54 medicinal species, of which 33 are commercialized [82]. Additionally, some botanical species traditionally harvested in Brazil, such as Arnica (*Lychnophora ericoides *Mart., 1982) and Jaborandi (genus *Pilocarpus*) [83] figure on the National list of endangered flora. Moreover, some species without official protection are being subject to a strong harvesting pressure, as in the case of the Espinheira-santa (*Maytenus ilicifolia *Mart.ex Reiss.) [84].

## Sanitary Concerns

Traditional drugs and traditional medicine in general represent a still poorly explored field of research in terms of therapeutic potential or clinical evaluation. There is a current preoccupation about this, since it is well-established that all sorts of vegetable, animal and mineral remedies used in a traditional setting are capable of producing serious adverse reactions. It is essential, however, that traditional drug therapies be submitted to an appropriate benefit/risk analysis [85].

Plants have an advantage in this area based on their long-term use by humans (often hundreds or thousands of years). One might expect any bioactive compounds obtained from such plants to have low human toxicity. Obviously, some of these plants may be toxic within a given endemic culture that has no reporting system to document these effects. It is unlikely, however, that acute toxic effects following the use of a plant in these cultures would not be noticed, and the plant would then be used cautiously or not at all. Chronic toxic effects would be less likely to signal that the plant should not be used. In addition, chemical diversity of secondary plant metabolites resulting from plant evolution may be equal or superior to that found in synthetic combinatorial chemical libraries [86].

Numerous case reports and case series of heavy metal poisoning associated with the use of traditional Chinese medicines (TCMs) have been published [87]. WHO has emphasized the importance of scientific investigations into indigenous herbal medicines [88], and many source countries look upon native medicinal plants as possible additions to the WHO list of "essential drugs", once their value has been clinically proven.

It is known, however, that numerous infectious diseases can be transmitted from animals to humans (i.e. zoonoses). In this context, the possibility of transmitting infections or ailments from animal preparations to the patient should be seriously considered [89]. Several organs and tissues including bones and bile can be a source of *Salmonella *infection causing chronic diarrhoea and endotoxic shock. The possibility of transmission of other serious and widespread zoonoses such as tuberculosis or rabies should be considered whenever animal tissues from unknown sources are handled and used as remedies [90]. The possibility of toxic or allergic reactions to animal products should also be considered.

Nevertheless, a study conducted in NE Brazil has shown that most users of animal products as medicines had a perception that almost none of the remedies had adverse side effects unless the dosage and administration were inappropriate [80]. This example clearly illustrates the need to further explore possibilities that would foster successful integration of TM into a public health framework. Educational programs for stakeholders could maximize benefits of TM, while reducing risks to users.

## Conclusion

The interdependence between the sustainability of the environment and the sustainability of the human species needs full recognition and the development of new public health practices [91], which can increasingly translate into policies and actions the recognition that the sustainable use of finite natural resources is a major determinant of health.

It is well established that TM plays a crucial role in health care for a large part of the population living in developing countries. In fact, for centuries, TM was the only health care system available to the prevention and treatment of diseases in different cultures. The interfaces among public health, TM and biodiversity conservation encompass a number of relevant and contemporary issues which are becoming increasingly apparent, as exemplified by WHO's goal in medicines: "to help save lives and improve health by ensuring the quality, efficacy, safety and rational use of medicines, including traditional medicines, and by promoting equitable and sustainable access to essential medicines, particularly for the poor and disadvantaged".

The formal recognition and respect that major traditional medicinal systems around the world are gaining [92], allied to the extensive practice of traditional medicine in developing countries and the rapidly growing demand for alternative and basic therapeutic means (also in industrialized countries) constitute the international relevancy of research and development in the field of traditional drugs [93]. Moreover, there is a growing recognition that knowledge of TM is important not only because of its potential to discover new treatments, but also because of its socioeconomic, conservationist and cultural components. As pointed out by Bodeker and Kronenberg [94], public health researchers must lead the development of a research agenda that considers social, cultural, political and economic contexts, to maximize the potential contribution of TM to healthcare systems globally.

The consequences for human well-being and health of disruptions to ecosystems are much more diverse and remain largely unstudied. It is therefore difficult to quantify current and future health effects of biodiversity losses and other changes to ecosystems. We are, however, acquiring new understanding of how the processes of forest clearance, agricultural practice, animal husbandry, river dams, and irrigation systems affect the emergence or the geographic and seasonal range of infectious diseases in humans [28].

Given the increased use of TM, possibilities that would ensure successful integration of TM into a public health framework should be explored. Potential bilateral benefits, limitations, and, ultimately, roles that TM and biomedical approaches may assume within an integrative system of care should be better illustrated [95].

The construction of a broad public health agenda is in order. Such agenda should evolve with an awareness of social, cultural, and political dimensions and should address values (equity, ethics), sustainability (regulation, financing, knowledge generation, knowledge management, capacity building), and the research environment [94].

The construction of regulatory measures will increasingly require the involvement of stakeholders, who must be made aware of the need for the conservation of the natural resource as a guarantee for its sustainable exploitation. Such involvement, besides contributing to the construction of direct conservation measures and of the proposition of feasible management options, perhaps could contribute to change the perception held by some that the demands for regulation to protect endangered species represent a form of cultural imperialism.

In that same direction, informed participation of holders of traditional medical knowledge in consultation/decision processes may further foster much needed co-operation to ensure the equitable sharing of the benefits arising from the utilization of traditional knowledge, innovations and practices [94].

## Declaration of competing interests

The author(s) declare that they have no competing interests.

## References

[B1] WHO World Health Organization Traditional medicine strategy 2002–2005 2002 http://whqlibdoc.who.int/hq/2002/WHO_EDM_TRM_2002.1.pdf

[B2] Good C Meade MS Ethno-medical Systems in Africa and the LDCs: Key Issues in Medical Geography Conceptual and Methodological Issues in Medical Geography 1980 University of North Carolina at Chapel Hill, Studies in Geography 93 116

[B3] Gesler WM Therapeutic landscapes: medical Issues in Light of the new cultural geography Social Science & Medicine 1992 34 7 735 746 10.1016/0277-9536(92)90360-3 1376497

[B4] Millennium Ecosystem Assessment Ecosystems and Human Well-being: Synthesis 2005 Island Press, Washington, DC

[B5] Anyinam C Ecology and Ethnomedicine: Exploring Links Between Current Environmental Crisis and Indigenous Medical Practices Social Science & Medicine 1995 40 3 321 329 10.1016/0277-9536(94)E0098-D 7899944

[B6] Alves RRN Rosa IL Why study the use of animal products in traditional medicines? Journal of Ethnobiology and Ethnomedicine 2005 1 1 5 10.1186/1746-4269-1-1 16270931 PMC1277085

[B7] McMichael AJ Beaglehole R The changing global context of public health Lancet 2000 356 495 499 10.1016/S0140-6736(00)02564-2 10981904

[B8] Lebel J Health: an ecosystem approach 2003 International Development Research Centre

[B9] Pushpangadan P Behl HM Environment & Biodiversity: Agenda for future ICPEP-3 2005 http://www.geocities.com/isebindia/ICPEP-3/ICPEP3-S-2.html

[B10] Chivian E Biodiversity: Its Importance to Human Health 2002 Center for Health and the Global Environment. Harvard Medical School

[B11] Cowden CJ Paterson I Cancer drugs better than taxol? Nature 1997 387 238 239 10.1038/387238a0 9153384

[B12] Nicolaou KC Guy RK Potier P Taxoids: new weapons against cancer Scientific American 1996 274 6 94 8 8643952 10.1038/scientificamerican0696-94

[B13] McGuire WP Rowinsky EK Rosenshein NB Grumbine FC Ettinger DS Armstrong DK Donehower RC Taxol: a unique antineoplastic agent with significant activity in advanced ovarian epithelial neoplasms Ann Intern Med 1989 111 4 273 9 2569287 10.7326/0003-4819-111-4-273

[B14] Olivera BM Rivier J Clark C Ramilo CA Corpuz GP Abogadie FC Mena EE Woodward SR Hillyard DR Cruz LJ Diversity of *Conus *neuropeptides Science 1990 249 4966 257 263 10.1126/science.2165278 2165278

[B15] Malmberg AB Yaksh TL Effects of continuous intrathecal infusion of omega-conopeptides on behavior and anti-nociception in the formalin and hot plate tests in rats Pain 1994 60 83 90 10.1016/0304-3959(94)00094-U 7715945

[B16] Valentino K Newcomb R Gadbois T Singh T Bowersox S Bitner S Justice A Yamashiro D Hoffman BB Ciaranello R Miljanich G Ramachandran J A selective N-type calcium channel antagonist protects against neuronal loss after global cerebral ischemia Proc Natl Acad Sci 1993 90 7894 7 10.1073/pnas.90.16.7894 8102803 PMC47249

[B17] Miljanich GP Venom peptides as human pharmaceuticals Sci Med 1997 4 5 6 15

[B18] Bowersox SS Tich N Mayo M Luther R SNX-111: antinociceptive agent Ntype voltage-sensitive calcium channel blocker Drugs Future 1998 23 2 152 60 10.1358/dof.1998.023.02.443399

[B19] Jones GP McCormick MI Srinivasan M Eagle JV Coral decline threatens fish biodiversity in marine reserves PNAS 2004 101 8251 8253 10.1073/pnas.0401277101 15150414 PMC419589

[B20] Anderson PK Morales FJ The emergence of new plant diseases: the case of insect- transmitted plant viruses Ann N Y Acad Sci 1994 740 181 94 10.1111/j.1749-6632.1994.tb19868.x 7840449

[B21] Epstein PR Climate, ecology, and human health Consequences 1997 3 2 2 19

[B22] Daszak P Cunningham AA Hyatt AD Emerging infectious diseases of wildlife: threats to biodiversity and human health Science 2000 287 443 9 10.1126/science.287.5452.443 10642539

[B23] Ostfeld RS Keesing F Biodiversity and disease risk: the case of Lyme disease Conserv Biology 2000 14 3 722 8 10.1046/j.1523-1739.2000.99014.x

[B24] Walsh JF Molyneux DH Birley MH Deforestation: effects on vector-borne disease Parasitology 1993 106 S55 S75 8488073 10.1017/s0031182000086121

[B25] Ostfeld RS Keesing F Jones CG Canham CD Lovett GM Integrative ecology and the dynamics of species in oak forests Integr Biol 1998 1 178 86 10.1002/(SICI)1520-6602(1998)1:5<178::AID-INBI3>3.0.CO;2-C

[B26] Morse SS Factors in the emergence of infectious diseases Emerg Infect Dis 1995 1 1 7 15 8903148 10.3201/eid0101.950102 PMC2626828

[B27] Wenzel RP A new hantavirus infection in North America N Engl J Med 1994 330 14 1004 5 10.1056/NEJM199404073301410 8121440

[B28] McMichael AJ Woodruff RE Detecting the Health Effects of Environmental Change: Scientific and Political Challenge EcoHealth 2005 2 1 3 10.1007/s10393-004-0152-0

[B29] Reiter P Climate Change and Mosquito-Borne Disease Environ Health Persp 2001 1 141 161 10.2307/3434853 PMC1240549 11250812

[B30] Bell D Robertson S Hunter PR Animal origins of SARS coronavirus: possible links with the international trade in small carnivores Phil Trans R Soc Lond 2004 359 1107 14 10.1098/rstb.2004.1492 15306396 PMC1693393

[B31] Shanley P Luz L The impacts of forest degradation on medicinal plant use and implications for health care in eastern Amazonia BioScience 2003 53 6 573 584 10.1641/0006-3568(2003)053[0573:TIOFDO]2.0.CO;2

[B32] Daily GC Ehrlich PR Global change and human susceptibility to diseases Annu Rev Energy Environ 1996 21 125 44 10.1146/annurev.energy.21.1.125

[B33] Calixto JB Twenty-five years of research on medicinal plants in Latin America J Ethnopharmacol 2005 100 131 134 10.1016/j.jep.2005.06.004 16006081

[B34] Kerharo J Traditional Pharmacopoieas and Environment African Environ 1975 1 30

[B35] Anyinam CA Persistence with Change: A Rural-Urban Study of Ethno-Medical Practices in Contemporary Ghana PhD thesis 1987 Queen's University, Kingston, Ontario

[B36] Castello L A Method to Count Pirarucu *Arapaima gigas*: Fishers, Assessment and Management North American J Fish Managmt 2004 24 379 389 10.1577/M02-024.1

[B37] Gillingham S Social Organization and Participatory Resource Management in Brazilian Ribeirinho Communities: A Case Study of the Mamirauá Sustainable Development Reserve, Amazonas Society & Natural Resources 2001 14 9 803 814 10.1080/089419201753210611

[B38] Child B The practice and principles of community-based wildlife management in Zimbabwe: the CAMPFIRE programme Biodiversity and Conservation 1996 5 3 369 398 10.1007/BF00051780

[B39] Pomeroy RS Carlos MB Community-based coastal resource management in the Philippines: a review and evaluation of programs and projects, 1984–1994 Marine Policy 1997 21 5 445 464 10.1016/S0308-597X(97)00016-X

[B40] Michael K Lambeth L Fisheries management by communities: a manual on promoting the management of subsistence fisheries by Pacific Island communities 2000 Secretariat of the Pacific Community

[B41] Lev E Traditional healing with animals (zootherapy): medieval to present-day Levantine practice J Ethnopharmacol 2003 86 107 118 10.1016/S0378-8741(02)00377-X 12576209

[B42] Yesilada E Past and future contributions to traditional medicine in the health care system of the Middle-East J Ethnopharmacol 2005 100 1–2 135 137 10.1016/j.jep.2005.06.003 15994043

[B43] Alves RRN Rosa IL From cnidarians to mammals: The use of animals as remedies in fishing communities in NE Brazil Journal of Ethnopharmacology 2006 107 259 276 10.1016/j.jep.2006.03.007 16621379

[B44] Adeola MO Importance of wild Animals and their parts in the culture, religious festivals, and traditional medicine, of Nigeria Environ Conserv 1992 19 2 125 134

[B45] Farnsworth NR Morris RW Higher plants: the sleeping giant for drug development Am J Pharm 1976 148 46 52 984178

[B46] Farnsworth NR Soejarto DD Potential consequence of plant extinction in the United States on the current and future availability of prescription drugs Econ Bot 1985 39 3 231 40

[B47] Soejarto DD Biodiversity prospecting and benefit-sharing: perspectives from the field J Ethnopharmacol 1996 51 1 15 10.1016/0378-8741(95)01345-8 9213606

[B48] Grifo F Rosenthal J Biodiversity and Human Health 1997 Island Press, Washington, D.C.

[B49] Grifo F Newman D Fairfield AS Grifo F, Rosenthal J The origins of prescription drugs Biodiversity and human health 1997 Washington, DC: Island Press 131 163

[B50] Chivian E Environment and health: 7. Species loss and ecosystem disruption – the implications for human health CMAJ 2001 164 1 66 9 11202670 PMC80637

[B51] Center for Biodiversity and Conservation (CBC) Biodiversity and Human Health: A Guide for Policymakers 1997 New York, NY: American Museum of Natural History http://research.amnh.org/biodiversity/acrobat/policy.pdf

[B52] Chivian E Grifo F, Rosenthal J Global environmental degradation and species loss: implications for human health Biodiversity and Human Health 1997 Washington: Island Press 7 38

[B53] Farnsworth NR Bingel AS Wagner H, Wolff P Problems and prospects of discovering new drugs from higher plants by pharmacological screening New Natural Products and Plant Drugs with Pharmacological, Biological or Therapeutic Activity 1997 Berlin: Springer 1 22

[B54] Farnsworth NR Wilson EO Screening plants for new medicines Biodiversity 1988 Washington, D.C.: National Academy Press 83 97

[B55] Balick MJ Anonymous Ethnobotany and the identification of therapeutic agents from the rainforest Bioactive Compounds from Plants 1990 Ciba Foundation Symposium 154. Wiley Interscience, New York 22 31 10.1002/9780470514009.ch3 2086039

[B56] Cox PA Anonymous Ethnopharmacology and the search for new drugs Bioactive Compounds from Plants 1990 Ciba Foundation Symposium 154. Wiley Interscience, New York 40 47

[B57] Farnsworth NR Anonymous The role of ethnopharmacology in drug development Bioactive Compounds from Plants 1990 Ciba Foundation Symposium 154. Wiley Interscience, New York 2 11 10.1002/9780470514009.ch2 2086037

[B58] Cox PA Balick MJ The ethnobotanical approach to drug discovery Scientific American 1994 270 6 82 87 8023119

[B59] Kunin WE Lawton JH Gaston KJ Does biodiversity matter? Evaluating the case for conserving species Biodiversity: a biology of numbers and differences 1996 Blackwell Science 283 308

[B60] Marques JGW Fauna medicinal: Recurso do ambiente ou ameaça à biodiversidade? Mutum 1997 1 1 4

[B61] World Resources Institute World Resources Report 2000–2001. People and ecosystems: the fraying web of life 2000 Washington D.C.: World Resources Institute

[B62] Bisset NG One man's poison, another man's medicine? Journal of Ethnopharmacology 1991 32 71 81 10.1016/0378-8741(91)90105-M 1881170

[B63] Carté BK Biomedical potential of marine natural products BioScience 1996 46 271 286 10.2307/1312834

[B64] Faulkner DJ Biomedical uses for natural marine chemicals Oceanus 1992 35 29 35

[B65] Kang S Phipps M A question of attitude: South Korea's Traditional Medicine Practitioners and Wildlife Conservation 2003 TRAFFIC East Asia, Hong Kong

[B66] Correa CM Protection and promotion of traditional medicine: implications for public health in developing countries 2002 Switzerland: South Centre

[B67] Lettington R The protection and Promotion of Traditional and Marginalized Medicinal knowledge: Romantic Dream or Twenty-First century Necessity? Regional Conference on medicinal Plants, Traditional Medicines and Local Communities in Africa: Challenges and opportunities of the New Millenium, Nairobi 2000

[B68] Ten Kate K Laird S The Commercial Use of Biodiversity: Access to Genetic Resources and Benefit-Sharing 1999 Commission of the European Communities and Earthscan Publications Ltd. London

[B69] Hamilton A Medicinal Plants and Conservation: Issues and Approaches 2003 WWF UK report

[B70] Prance GT An ethnobotanical comparison of four tribes of Amazonian indians Acta Amazônica 1972 2 2 7 27

[B71] Balée W A Etnobotânica Quantitativa dos Índios Tembé Boletin Museu Paraense 1986 3 29 50

[B72] Balée W Análise Preliminar de Inventário Florestal e a Etnobotânica Ka'apor. Boletin Museu Paraense 1987 3 29 50

[B73] Branch LC Silva MF Folk medicine of Alter do Chão, Pará, Brazil Acta Amazônica 1983 13 5 6

[B74] Amorozo MCM Gély A Uso de plantas medicinais por caboclos do Baixo Amazonas, Barcarena, PA. Brasil Boletin de Museu Paraense 1988 1 47 131

[B75] Voeks RA Tropical forest healers and habitat preference Economic Botany 1996 50 381 400

[B76] Stepp JR Moerman DE The importance of weeds in ethnopharmacology Journal of Ethnopharmacology 2001 75 19 23 10.1016/S0378-8741(00)00385-8 11282438

[B77] Fowler C Hodgkin T Plant genetic resources for food and agriculture: assessing global availability Annu Rev Environ Resour 2004 29 143 79 10.1146/annurev.energy.29.062403.102203

[B78] Beinart W Middleton K Plant transfer in historical perspective: A review article Environ Hist 2004 10 3 29

[B79] Voeks RA Disturbance pharmacopeias: Medicine and myth from the humid tropics Ann Assoc Am Geogr 2004 94 4 868 888

[B80] Alves RRN Rosa IL Zootherapeutic practices among fishing communities in North and Northeast Brazil: A comparison Journal of Ethnopharmacology in press doi:10.1016/j.jep.2006.10.033 10.1016/j.jep.2006.10.033 17118592

[B81] Sodeinde OA Soewu DA Pilot study of the traditional medicine trade in Nigeria Traffic Bulletin 1999 18 1 35 40

[B82] Silva SR Buitrón X Oliveira LH Martins MVM Plantas medicinais doBrasil: aspectos gerais sobre legislação e comércio 2001 Ministério de Cooperação Econômica e Desenvolvimento da Alemanha & IBAMA. Relatório Final

[B83] SBB – Sociedade brasileira de Botânica Centuria Plantarum Brasiliensium Exstintionis Minitata 1992 Brasília, DF

[B84] Silva SR Ediviges, Mineiros, GO Uma nota sobre a Exploração de Plantas Medicinais do Cerrado Plantas Medicinais do Cerrado: Perspectivas comunitárias para a saúde, o meio ambiente e o desenvolvimento sustentável 1999 237 245

[B85] De Smet PAGM Is there any danger in using traditional remedies? J Ethnopharmacol 1991 32 43 50 10.1016/0378-8741(91)90102-J 1881166

[B86] Fabricant DS Farnsworth NR The Value of Plants Used in Traditional Medicine for Drug Discovery Environ Health Persp 2001 1 69 75 10.2307/3434847 PMC1240543 11250806

[B87] Ernst E Thompson Coon J Heavy metals in traditional Chinese medicines: a systematic review Clin Pharmacol Ther 2001 70 497 504 11753265 10.1067/mcp.2001.120249

[B88] WHO (World Health Organization) The promotion and development of traditional medicine Technical Report Series, 622, WHO, Geneva 1978 102084

[B89] Still J Use of animal products in traditional Chinese medicine: environmental impact and health hazards Complement Ther Med 2003 11 118 122 10.1016/S0965-2299(03)00055-4 12801499

[B90] Schnurrenberger PR Hubbert WT An outline of the zoonoses 1981 Ames, IA: Iowa State University Press

[B91] Brown V Grootjans J Ritchie J Townsend M Verinder G Sustainability and health: supporting global ecological integrity in public health 2005 Earthscan Publications

[B92] Lee SKH Trade in traditional medicine using endangered species: an international context Proceedings of the second Australian Symposium on traditional medicine and wildlife conservation 1999 Melbourne Australia

[B93] Labadie RP Problems and possibilities in the use of traditional drugs J Ethnopharmacol 1986 15 221 230 10.1016/0378-8741(86)90162-5 3523048

[B94] Bodeker G Kronenberg F A Public Health Agenda for Complementary, Alternative and Traditional (indigenous) Medicine American Journal of Public Health 2002 92 10 1582 1591 12356597 10.2105/ajph.92.10.1582 PMC3221447

[B95] Giordano J Garcia MK Strickland G Integrating Chinese Traditional Medicine into a U.S. Public Health Paradigm J Altern Complem Med 2004 10 4 706 710 10.1089/acm.2004.10.706 15353031

